# Application of Iron Tailings-Based Composite Supplementary Cementitious Materials (SCMs) in Green Concrete

**DOI:** 10.3390/ma15113866

**Published:** 2022-05-28

**Authors:** Yannian Zhang, Daokui Yang, Xiaowei Gu, Hao Chen, Zhijun Li

**Affiliations:** 1 School of Civil Engineering, Shenyang Jianzhu University, Shenyang 110168, China; zyntiger@163.com (Y.Z.); ydk257200@outlook.com (D.Y.); ch961113@126.com (H.C.); 2 Science and Technology Innovation Center of Smart Water and Resource Environment, Northeastern University, Shenyang 110819, China; guxiaowei@mail.neu.edu.cn

**Keywords:** solid waste resourcing, ternary system, iron ore tailings, phosphate slag, steel slag, pore structure, interface transition zone, compressive strength

## Abstract

How to treat the iron tailings of mining solid waste with high value is an urgent problem on a global scale. In recent years, the application of iron tailings in the building materials industry has attracted the attention of many scholars. The conversion of iron tailings into green building materials helps achieve carbon neutrality and high-value utilization of solid waste, and promotes sustainable development. Although iron tailings have been extensively studied as supplementary cementitious materials, the performance of concrete is not ideal due to its low activity. In this study, the hybrid supplementary cementitious materials system was prepared by iron tailings, phosphorus slag, and steel slag, and the effects of supplementary cementitious materials type, iron tailings content, iron tailings grinding time, and supplementary cementitious materials content on concrete performance were studied. The compressive properties, iron tailings properties, pore structure, interfacial transition zone, and element distribution of hydration products of concrete were tested by compressive strength tests, X-ray Diffractometer (XRD), X-ray Photoelectron Spectroscopy (XPS), Mercury Intrusion Porosimetry (MIP), Backscattering Electron Tests (BSE), and Energy Dispersive Spectrometer (EDS). The results show that further grinding improves the iron tailings activity. There is a synergistic mechanism between steel slag and phosphorus slag in the composite supplementary cementitious materials, which overcomes the low activity defect of iron tailings and produces concrete with a compressive strength exceeding 40 MPa. The composite supplementary cementitious materials can optimize the interfacial transition zone of the concrete interface and reduce the calcium–silicon ratio of the hydration products. However, it will deteriorate the pore structure of the concrete matrix, cause part of the concrete matrix to be damaged and lead to a loss of compressive strength, and the loss is acceptable. This work broadens the methods of comprehensive utilization of iron tailings and also provides a reference for a more detailed understanding of the properties of iron tailings-based concrete.

## 1. Introduction

The continuous development of mineral resources forms many tailings, which is one of the rich solid waste resources. The accumulation of tailings pollutes the environment and poses a threat to the safety of surrounding people and buildings [1,2]. Iron tailings (IOTs) account for the most significant proportion of the total tailings’ generation in China: about 520 million tons, accounting for 40.9% of the total tailings’ generation [3,4]. With extensive research on the recycling of IOTs at home and abroad, IOTs are now often used for the recovery of metals from secondary resources [5], backfill materials [6,7], concrete coarse and fine aggregates [8,9], and bricks [10,11].

Due to the presence of SiO_2_ and Al_2_O_3_ in IOTs, many scholars have tried to prepare supplementary cementitious materials (SCMs) using IOTs [12,13]. The majority of the SCMs are silica-aluminous volcanic materials, which are widely used in concrete and are thought to be an effective way to improve cement performance while lowering cement costs [14,15]. The interfacial transition zone (ITZ) is often considered the weak link in cementitious composites, which largely affects the mechanical properties and durability of concrete [16]. Mixing auxiliary cementing materials, such as slag, fly ash, and silica fume, can improve the structure of the ITZ [17]. SCMs can fill the pores by generating cementing substances through volcanic ash reactions, reducing the wall effect and percolation reaction, and improving the pore structure of the ITZ [18,19].

The mineral composition of IOTs is mostly inactive crystalline silica, so some means will be used to activate the IOTs, and currently, the most commonly used means is mechanical activation [20]. It was found that the physical and chemical properties of solid particles are changed to some extent under the action of mechanical forces, resulting in a decrease in size, an increase in specific surface area, and an increase in chemical activity [21,22]. However, mechanically activated IOTs still belong to low active volcanic ash materials [23,24], and when less than 10% admixture of active IOTs was used to prepare concrete, the strength of the test piece only reaches 30 MPa [25]. It was found that slag or fly ash (FA) can replace some of the IOTs to produce precast concrete, which can make the precast concrete perform well [26,27]. The proposed composite SCMs system has important reference significance for the comprehensive utilization of IOTs. With the acceleration of the urbanization construction process, the demand for SCMs in the construction industry has gradually increased. The price of traditional highly active SCMs, such as slag powder, fly ash, and silica fume, has increased, and the reserves are limited. Therefore, more composite SCMs systems need to be developed to achieve the purpose of IOTs elimination through the use of higher activity SCMs and IOTs compounding.

Phosphorus slag (PS) is a by-product produced in the process of high-temperature electric furnace phosphorus removal. China is the second-largest country globally with abundant phosphorus reserves and a significant producer and exporter of yellow phosphorus, with an annual output of more than 8 million tons [28,29]. The main components of PS are SiO_2_ and CaO, and the content of the glass phase can reach 85~90%, which is similar to ground granulated blast furnace slag (GGBFS) and has particular potential activity [28,30,31]. The hydration reaction of PS generates additional C-S-H gel, which increases the density of the structure and reduces the number of harmful pores [28,32]. At the same time, PS can reduce the hydration heat of the reaction and has good durability [33]. However, PS containing soluble P and F is not conducive to the early hydration of cement [34,35], and steel slag can be added to eliminate the adverse effects of low early strength and retardation [36].

Steel slag (SS) is a by-product of the steelmaking process, and the annual output of SS in China reaches 100 million tons [37]. Since the mineral phase of SS is mainly C_2_S, followed by C_3_S, it is considered a potential cementitious material [38,39]. However, the utilization rate of SS in China is only 29.5%, and the effective utilization of SS is also a vital issue.

In summary, it is potentially feasible to prepare a composite SCMs system using PS, SS, and IOTs. In this study, based on mechanical grinding of IOTs and also to maximize the use of IOTs, natural sand and gravel aggregates were prepared by replacing natural sand and gravel aggregates with IOTs sand and iron ore waste rock. The effects of the type of auxiliary cementing material, IOTs admixture, IOTs grinding time, and the admixture of auxiliary cementing material on the compressive properties of concrete were analyzed. The pore structure, ITZ, and the calcium–silica ratio of the ITZ of concrete were tested using MIP, BSE, and EDS, respectively. Further, the relationship between the compressive properties and the properties of the pore structure and ITZ was investigated. It provides a reference for the development of auxiliary cementing materials for composite systems and the utilization of IOTs in the full particle size range.

## 2. Experimental

### 2.1. Raw Materials

**Figure 1:**
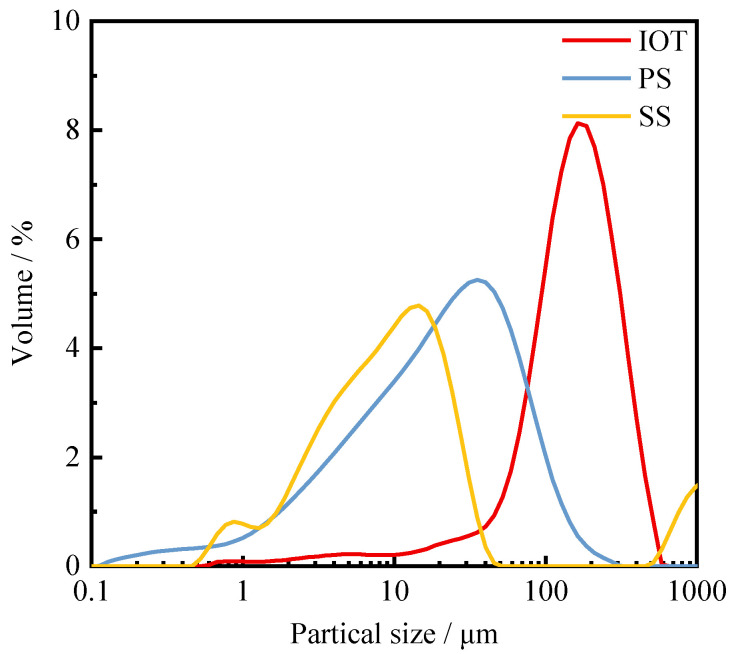
Particle size distributions of IOT, PS, and SS.

**Figure 2:**
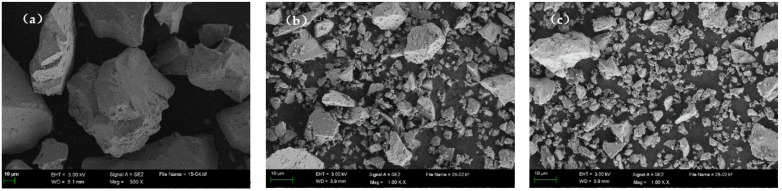
SEM images of (**a**) IOTs, (**b**)PS, and (**c**)SS.

**Figure 3:**
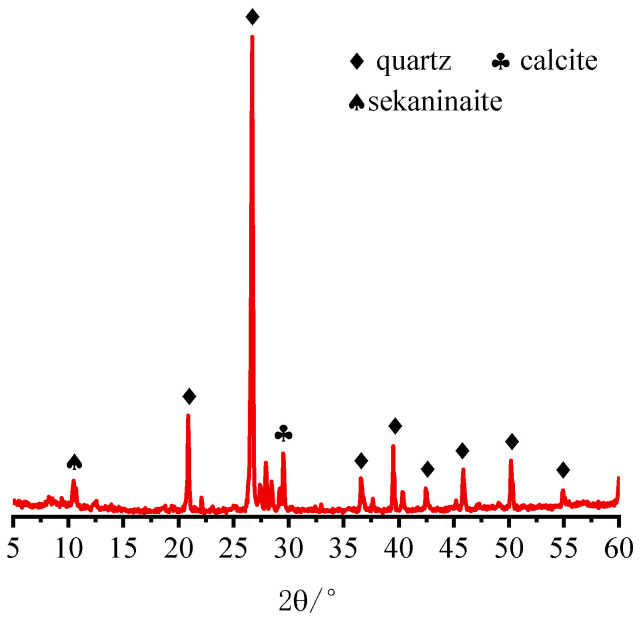
The XRD pattern of the IOT.

IOTs, PS, and SS are used as SCMs to prepare concrete. IOTs are obtained from Waitou Mountain in Liaoning Province, China. PS is produced in Yunnan Province, China. SS is sourced from Shanghai, China. P·O 42.5 Portland cement complying with the Chinese National Standard GB175-2007 [40]. Coarse aggregates (IOTs-CA) and fine aggregates (IOTs-FA) for preparing concrete were taken from Liaoning Yilifang Sand Industry Co., Ltd (Shenyang, China). The fineness modulus of fine aggregates was 2.0, and the size range of coarse aggregates was 5–20 mm. The P-II water reducer (WR) used in this study was produced by Shenyang Shengxinyuan Building Materials Co., Ltd (Shenyang, China). The chemical composition of IOTs, PS, SS, and cement determined by X-ray fluorescence (XRF) analysis is shown in Table 1. The particle size distribution of IOTs, PS, and SS is shown in Figure 1, and SEM micrographs are shown in Figure 2. The IOTs determined by X-ray diffraction (XRD) analysis are shown in Figure 3. It can be seen that the crystalline phase of IOTs is mainly composed of quartz, calcite, and iron cordierite.

### 2.2. Experimental Methods

#### 2.2.1. Grinding

The IOTs were dried in an oven at 105 °C. After drying, the IOTs were sieved and particles between 0.075–0.15 mm were selected for grinding to improve the crushing efficiency and reduce the effect of ultra-fine powders smaller than 0.075 mm on the crushing. The dried IOTs were ground in an XQM-4 laboratory ball mill (Tencan Powder, Changsha, China) for 1.5 h, 2 h, and 2.5 h, respectively. We used Micromeritics ASAP 2460 (Micromeritics, Shanghai, China) to determine the specific surface area and pore size distribution of the milled iron tailings, and nitrogen was selected as the adsorbent. For degassing, the temperature was increased to 90 °C for 1 h at 10 °C/min and then increased to 200 °C for 4 h at 10 °C/min. After degassing, the samples were cooled to room temperature, and the samples were taken out and tested in liquid nitrogen at −196 °C.

#### 2.2.2. X-ray Photoelectron Spectroscopy

X-ray photoelectron spectroscopy (XPS) was used to determine the surface binding energy of Al, Si, and Ca elements in IOTs at different grinding times. The test conditions were as follows: the vacuum of the chamber was 8.0–10 Pa, the excitation source was Al K-rays with energy of 1.4867 keV, and the flux energies of the full and fine spectra were 100 eV and 30 eV, respectively, in steps of 0.1 eV, with a dwell time of 40–50 ms, and the binding energy of C1s (284.8 eV) was used as the energy standard for charge correction.

#### 2.2.3. X-ray Diffractometer

A Rigaku Ultima IV X-ray diffractometer (Rigaku, Beijing, China) with CuKaα radiation (135 mA and 40 kV) was used to identify the main crystalline phases of the crushed IOTs. The IOTs were well ground before the experiment and an appropriate amount of samples was placed on a slide and flattened and compacted. Data were collected in 0.02° (instrumental) steps at a scanning speed of 3° per minute and a scanning angle of 5°–60° (2). Phase identification was performed using MDI Jade6 software.

#### 2.2.4. Preparation of Specimens

The concrete mix ratios are listed in Table 2. Concrete samples (M1, I1, G0, and C0) were used as reference samples. SCMs type (IOTs single blending (M1), IOTs-PS double blending (M2), IOTs-PS-SS triple blending (M3)), IOTs content (IOTs blended with 15% (I15), IOTs blended with 10.5% (I10.5), IOTs blended with 6% (I6), IOTs blended with 1.5% (I1.5)), IOTs grinding time (IOTs grinding 0 h (G0), IOTs grinding 1.5 h (G1.5), IOTs grinding 2 h (G2), IOTs grinding 2.5 h (G2.5)) and SCMs content (no SCMs mixed (C0), SCMs mixed with 10% (C10), SCMs mixed with 20% (C20), SCMs mixed with 30% (C30)), were used as variables. Where I6, G2, and C30 are the same sample, M3 and I15 is the same sample. Concretes of 100 × 100 × 100 mm were cast. Concretes were cured in a room with a temperature of 20 ± 1 °C and relative humidity higher than 95% (Standard curing condition).

#### 2.2.5. Compressive Strength

The compressive strength test of concrete is carried out concerning the “Standard for test methods of concrete physical and mechanical properties” (GB/T 50081-2019) [41]. The 7 d, 14 d, and 28 d compressive strengths of concrete were determined using a Shenzhen universal testing machine (2000 kN) with a loading rate of 0.7 MPa per second. The strength results were converted, and the conversion factor was 0.95. The test results were taken as the average of the strengths of the three specimens.

#### 2.2.6. Mercury Intrusion Porosimetry

To ensure the uniformity of the pore structure test and the concrete compressive performance test samples, the mercury compression test samples were taken from cubic concrete specimens after 28 days of curing. Firstly, a 15 mm thick slice of concrete was cut using a cutter parallel to the forming surface, and the plane at the cut was 15 mm away from the forming surface; then a core sample was drilled using an electric drill and a hollow drill bit (8–14 mm inner diameter of the drill bit), and the samples were all from the same depth of the cutting surface, and the sample did not contain aggregate. The sample does not need to ensure the uniformity of the rules and shape, and it is enough to ensure that no cracks are produced by external forces. After sampling, the samples were immersed in anhydrous ethanol for 7 days to terminate hydration, then dried for 3 days and tested for mercury compression (drying temperature of 50 ± 2 °C). The porosity and pore size distribution of the specimens were measured using an AutoPore IV 9510 fully automatic mercury piezometer (Micromeritics, Shanghai, China) with a maximum pressure of 414 MPa.

#### 2.2.7. Backscattering Electron Tests and Energy Dispersive Spectrometer

The specimens were sliced after curing for 28 days. The thickness of the slices was about 3–5 mm. The cutting direction was parallel to the forming surface. The first knife was 15 mm away from the forming surface, and the second knife was 18–20 mm away from the forming surface. Then the cores were drilled into the slices to take samples (containing aggregate and matrix). They were immersed in anhydrous ethanol for 7 days to terminate the hydration, and the samples were put into the oven to dry for 3 days (the drying temperature was 50 ± 2 °C). To prevent the samples to prevent the microstructure of the samples from being destroyed during the sample making process, the samples were immersed in epoxy resin for 24 h for natural hardening, followed by grinding, profiling, ultrasonic cleaning, and drying (the drying temperature was 50 ± 2 °C), and finally, the samples to be tested were obtained. The magnification of the backscattered images was 500 times, and the resolution of the images was 1024 × 768 Pixel. The images were quantified and calculated using image processing software (Image J 1.8.0).

The samples after BSE testing were further used for Energy dispersive spectrometer (EDS) testing. The calcium to silicon ratio of the product within the ITZ (100 μm from the aggregate) was determined by EDS, and three line scans were randomly performed for each sample.

## 3. Results and Discussion

### 3.1. Compressive Strength

**Figure 4:**
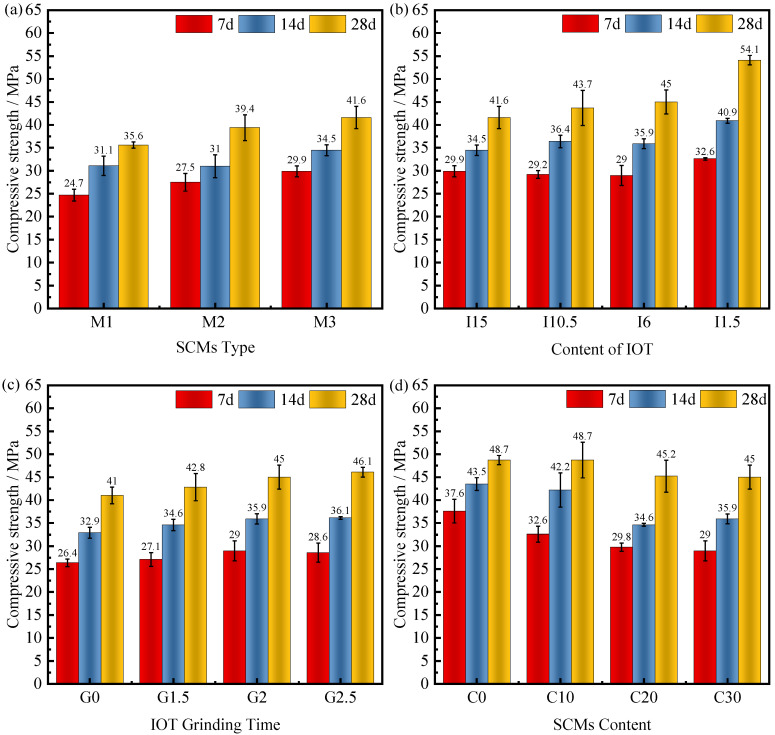
The influence of (**a**) SCMs Type, (**b**) Content of IOT, (**c**) IOT Grinding Time, and (**d**) SCMs Content on the compressive strength of concrete.

Figure 4a shows the effect of the type of SCMs on the compressive strength of concrete. The compressive strength of M2 increased by 11.3%, 6.9%, and 10.9% at 7, 14, and 28 days, respectively, compared with M1. Some studies have shown that PS itself has a high vitreous content and high volcanic ash activity [42]. Thus, the introduction of PS was able to produce more C-S-H gels compared to the IOTs system alone, thus improving the compressive strength of the concrete. This approach of compensating the deficiencies of one material with the advantages of another has been reported by several authors. Han et al. used slag powder to replace part of the IOTs to improve the performance of concrete [43]. Nano-silica can compensate for the loss of durability produced by fly ash on concrete [44].

Interestingly, the 7-day, 14-day, and 28-day compressive strengths of M3 increased by 8.7%, 10.3%, and 5.6%, respectively, compared to M2 after the incorporation of SS. SS admixture adds more CaO and promotes Ca(OH)_2_ crystallization [36]. Alkaline substances can better stimulate the secondary hydration reaction of PS, and SiO_2_ and Al_2_O_3_ are constantly dissolved in an alkaline environment to react with Ca(OH)_2_ to form C-S-H, improving compressive strength. As a result, the addition of the third component SS in the dopant stimulates the PS even more. The consumption of Ca(OH)_2_ by PS then facilitates the activity of SS, and the two form a synergistic mechanism.

To further analyze the effect of the synergistic mechanism between IOTs, SS, and PS on the compressive strength, the total amount of admixture was fixed at 30%, and the compressive performance of concrete was tested with the amount of IOTs admixture as a parameter. Figure 4b depicts the effect of IOTs admixture on concrete compressive strength. The trends of the 7-day and 14-day compressive strengths of concrete did not show any significant regularity. The 7-day compressive strengths of the left side of the boundary (I15 and I10.5) are close to those of the 6% IOTs admixture, and the 7-day compressive strengths of the right side of the boundary (I1.5) are significantly higher than those of the left side of the boundary. With the decrease in IOTs admixture, the overall 28-day compressive strength of concrete showed an increasing trend. This trend is the same as the pattern of change in compressive strength exhibited when IOTs are used as a single blend [45]. The above results show that the IOTs have a very low degree of reaction in the early stages and contribute negligibly to the compressive strength of concrete. It also proves that the synergistic mechanism of PS and SS can work in both early and late stages to significantly improve the compressive strength of concrete.

Studies have shown that IOTs mainly play a filling, nucleation, and dilution role in the cementitious material system. When the IOTs are fine, they can also play a certain volcanic ash activity [43]. Figure 4c demonstrates the effect of grinding time on the compressive strength of concrete. The compressive strength of concrete increased with the increase in grinding time of IOTs. This indicates that mechanical grinding facilitates the physical action and activity effect of IOTs, but the increase in concrete strength slows down after grinding time beyond 2 h. This is because the agglomeration effect occurs between the IOTs particles after grinding for more than 2 h, and the IOTs mainly play the filling effect in the SCMs. Although the IOTs activity is increasing under the action of mechanical force, the strength increase is not obvious due to the reduction of the filling effect [46].

The above results indicate the feasibility of using the synergistic mechanism between SS and PS to compensate for the low activity of IOTs. To compare the compressive performance difference between concrete containing ternary system admixtures and normal concrete and to analyze the reasons for it, the admixture amount of IOTs can be adjusted to 6% and the grinding time is preferably 2 h. Figure 4d shows the effect of the admixture of SCMs on the compressive strength of concrete, which gradually decreases with increasing admixture. The most significant difference is shown in the early stage, where the 7 d compressive strengths of C10, C20, and C30 decreased by 13.3%, 20.7%, and 22.9%, respectively, relative to C0. There are two main reasons as followed: 1. SS reduces the formation of AFt and inhibits the precipitation of CH and C–S–H [47]; 2. [PO4]^3−^ in the PS inhibits the formation of AFt, and [SO4]^2−^ ions prevent the conversion of “hexagonal hydrate” to C3AH6, and the early hydration of cement is inhibited [48]. The 28-day compressive strength of C10 remained the same as that of C0, and the 28 d compressive strength of C30 only decreased by 7.6% compared with that of C0, indicating that it is feasible to choose a 30% admixture of SCMs.

### 3.2. Mechanical Activation of IOT

**Figure 5:**
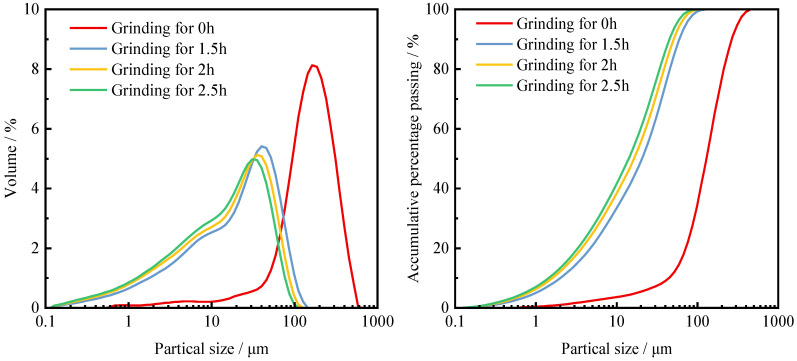
Particle size distribution and cumulative sieve allowance of IOTs at different grinding times.

The specific surface area of IOTs after grinding is shown in Table 3, and the particle size distribution and accumulated sieve allowance are shown in Figure 5. With the increase in grinding time, IOTs undergo brittle damage. Mechanical forces destroy their mineral structure, the particles become smaller, and the specific surface area increases rapidly [22,46]. The specific surface area of IOTs particles reached a peak of 1587 m^2^/kg after 2 h of mechanical grinding. As the particle size of IOTs particles became smaller, the material particles started to agglomerate under the effect of van der Waals forces. In this process, the free energy of the particles and the chemical potential energy of the system both decrease, so the material particles undergo an agglomeration phenomenon, and the specific surface area decreases [49]. Mechanical grinding produces fine particles of less than 1 μm in iron tailings particles. As the grinding time increases, the cumulative sieve residual of fine particles smaller than 1 μm increases. However, it is not possible to produce ultrafine powders smaller than 0.1 μm by grinding.

**Figure 6:**
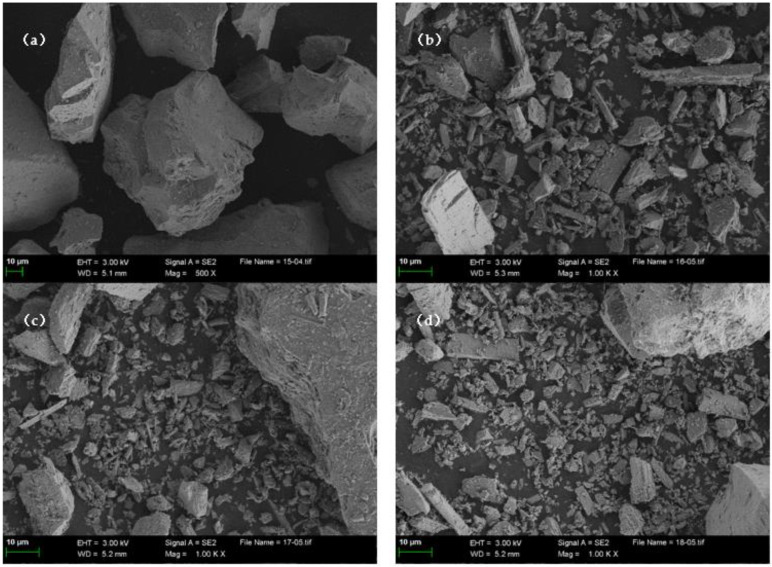
SEM images of (**a**) Grinding IOTs for 0 h, (**b**) Grinding IOTs for 1.5 h, (**c**) Grinding IOTs for 2 h, and (**d**) Grinding IOTs for 2 h.

The SEM micrographs of IOTs with different grinding times are shown in Figure 6. The original IOTs particles are irregular in shape, in the form of irregular polyhedra with many angles, with a relatively rough surface and no fine particles. After the mechanical grinding process, the IOTs particles with many internal cracks and defects are rapidly crushed, and the particles are gradually refined to appear as spherical particles, but most of them are still in the form of irregular polygonal polyhedra [49]. After grinding for more than 2 h, there is no significant change in particle size. Under the action of van der Waals forces, the particles agglomerate and the grinding efficiency becomes low [50]. IOTs with different grinding times showed similar morphology under SEM images, indicating that mechanical activation can only change the particle size of IOTs, but not the IOTs particle morphology.

**Figure 7:**
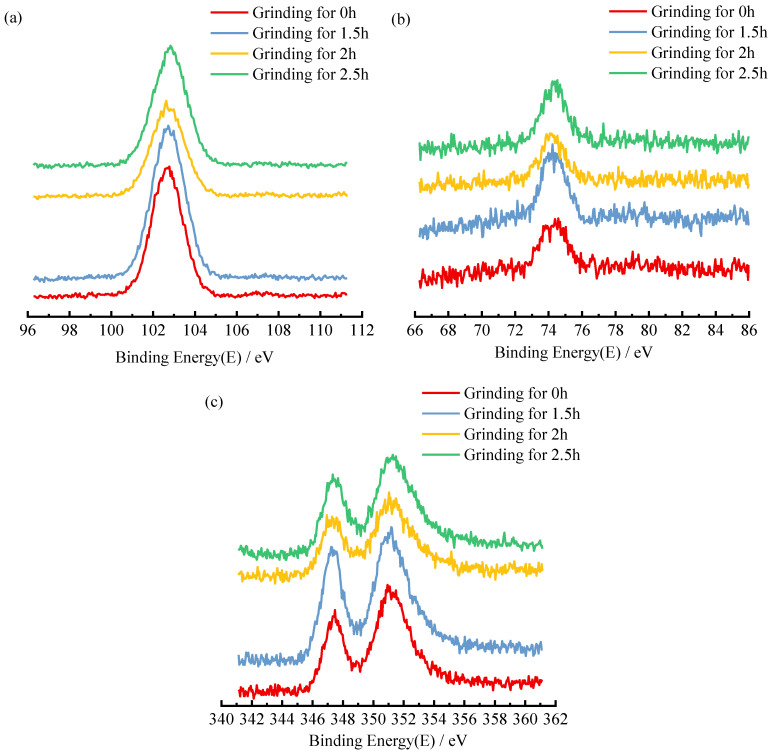
XPS patterns of IOTs at different grinding times of (**a**) binding energy of Si2p, (**b**) binding energy of Al2p, and (**c**) binding energy of Ca2p.

For inert silicate minerals such as IOTs, in which Al, Si, and Ca elements are present in the form of oxides, detecting the change of surface binding energy between Al, Si, and Ca elements and O elements can reflect the change pattern of the activity of alumina, silica, and calcium oxide in IOTs [51]. To determine the effect of mechanical grinding on the activity of IOTs, XPS analysis of IOTs powder with different grinding times was performed. Figure 7 shows the XPS energy spectra of IOTs powders with different grinding times. The surface binding energy of Al2p, Si2p, and Ca2p of mechanically activated IOTs decreased significantly and showed different trends compared with the original IOTs. Mechanical activation has a reduction effect on the surface binding energy of Al, Si, and Ca elements in IOTs, with a more significant reduction effect on Al elements. From the viewpoint of oxides, mechanical activation improves the reactivity of alumina, silica, and calcium oxide in IOTs by reducing the surface binding energy of the elements, with a significant increase in the activity of alumina. Despite the agglomeration effect of IOTs after grinding for more than 2 h, the activity of IOTs still increases as seen by XPS, which explains the continued increase in the intensity of IOTs grinding for 2.5 h.

**Figure 8:**
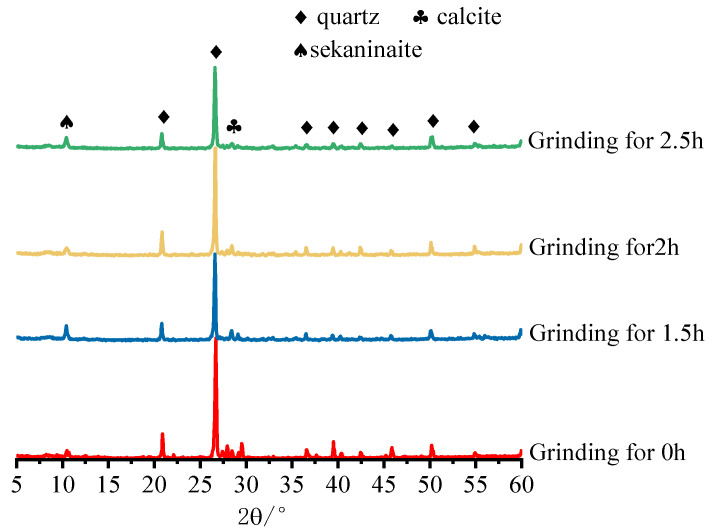
XRD patterns of IOTs at different grinding times.

To clarify the activation mechanism of IOTs by mechanical grinding, XRD spectra of IOTs at different grinding times were tested, and the results are shown in Figure 8. Compared with the original IOTs, the diffraction peak intensity of each phase of mechanically activated IOTs became lower, the crystallinity of SiO_2_ decreased, and the activity of IOTs was enhanced. This is because, during the mechanical grinding process, IOTs particles with lattice defects will undergo lattice distortion, increasing the plastic deformation of the particles and deepening the degree of amorphism, resulting in a decrease in the intensity of diffraction peaks [52,53]. The intensity of the diffraction peaks of each phase continued to decrease after grinding for more than 2 h, indicating that the activity of IOTs particles was still increasing, and thus the intensity of grinding for 2.5 h was still increasing.

### 3.3. Pore Structures

**Figure 9:**
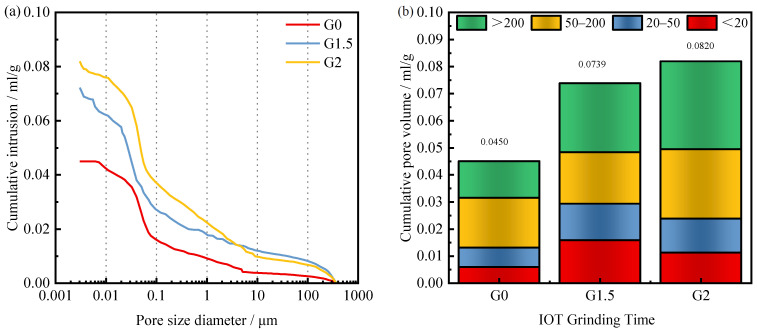
The influence of IOT grinding time on the (**a**) cumulative intrusion volume, and (**b**) pore volume distributions of the 28-d concrete.

Figure 9 shows the variation of the pore structure properties of the concrete matrix with the grinding time of IOTs. As shown in Figure 9a, the total pore volume of the concrete matrix increases as the IOTs grinding time increases. As mentioned earlier, IOTs can play the roles of filling, dilution, nucleation, and volcanic ash activity. As the fineness of IOTs increases, the role played should be more significant. However, the total pore volume of the concrete matrix showed an inverse trend. This result is consistent with the previous findings that the total porosity becomes larger with increasing fineness [54]. A similar trend appears in the pore size distribution test, as shown in Figure 9b, where the pore structure of the concrete matrix deteriorates as the grinding time of IOTs increases, i.e., the pores larger than 200 nm gradually increase. Although pores smaller than 20 nm were also increasing, the increase and the proportion of pores larger than 200 nm were much less than those larger than 200 nm.

**Figure 10:**
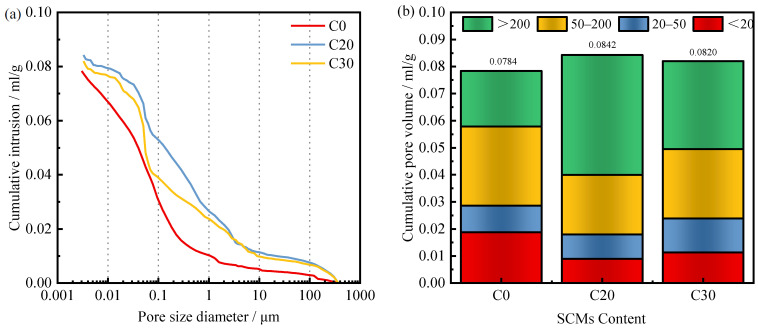
The influence of SCMs content on the (**a**) cumulative intrusion volume, and (**b**) pore volume distributions of the 28-d concrete.

Figure 10 shows the variation of the pore structure properties of the concrete matrix with the SCMs content. The total volume of pores in C20 and C30 is greater than that in C0, as shown in Figure 10a. As for the pore size distribution, it is shown in Figure 10b. The introduction of admixtures deteriorated the pore structure of the concrete matrix: the pores less than 20 nm in C0 were significantly higher than those in C20 and C30, and the pores larger than 200 nm were lower than those in C20 and C30.

There is significant inconsistency between the pore structure test results and the compressive strength test, G0 has better pore structure properties than G1.5 and G2, while the compressive resistance is the opposite. The pore structure is not the only factor that affects the compressive properties of concrete. The ITZ, as the weakest region in concrete, should be of concern. Therefore, the inconsistency between pore structure properties and compressive strength will be further discussed.

### 3.4. Interfacial Transition Zone

**Figure 11:**
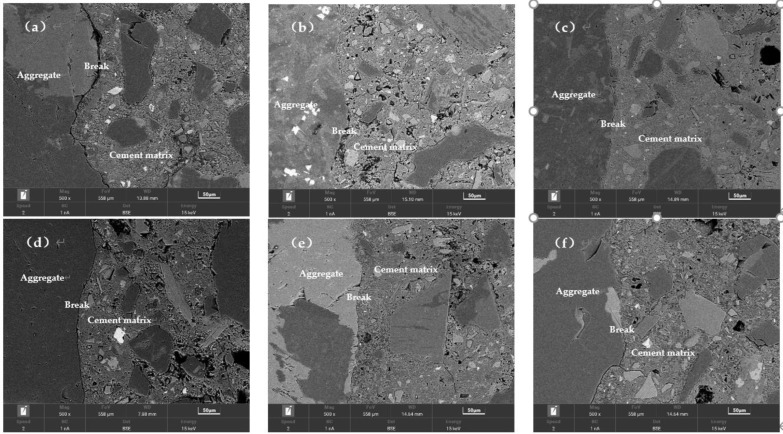
Identified BSE images of samples at 28-d of (**a**) G0, (**b**) G1.5, (**c**) G2, (**d**) C0, (**e**) C20, and (**f**) C30.

Figure 11 shows the BSE pictures of the 28-day concrete, which show the presence of unhydrated particles, micropores, and microcracks at the ITZ of each specimen. Compared with G0, the crack width at the ITZ of G1.5 and G2 is reduced and the matrix is denser. According to Figure 11d, there are more cracks at the ITZ when no SCMs are present, and the ITZ improves when SCMs are present.

**Figure 12:**
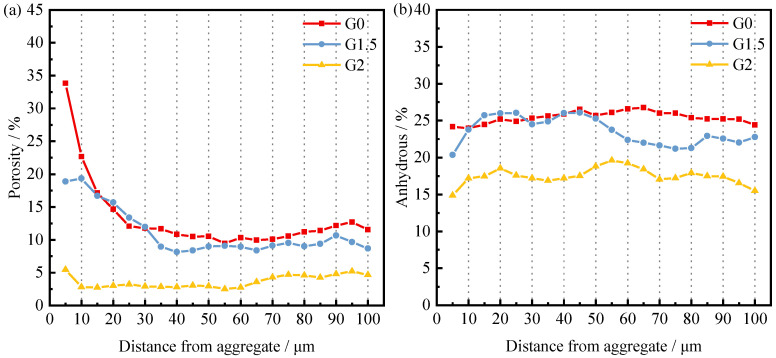
Effect of IOT grinding time on (**a**) Porosity, and (**b**) Anhydrous profiles of 28-d concrete.

**Figure 13:**
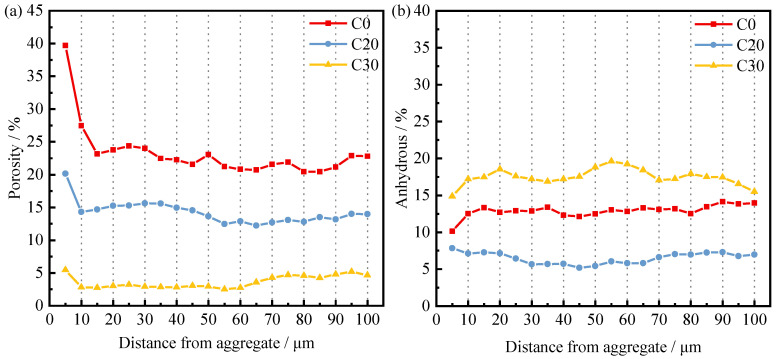
Effect of SCMs content on (**a**) Porosity, and (**b**) Anhydrous profiles of 28-d concrete.

To quantify the unhydrated particles and porosity near the transition zone at the interface of different concrete specimens, strip segmentation, and threshold segmentation were performed for the area within 100 μm of the aggregate, starting from the surface of the aggregate particles. The porosity and the percentage of the area corresponding to the unhydrated particles were determined for each segment using 5 μm as a section [55,56]. The result of the calculation is shown in Figure 12 and Figure 13.

Figure 12 shows the trend of porosity and unhydrated rate in the concrete ITZ with the grinding time of IOTs. Porosity increases from small to large in the order of G2, G1.5, and G0, as shown in Figure 12a. This indicates that extending the IOTs grinding time helps to reduce the porosity in the ITZ. Similarly, the variation of the unhydrated rate from low to high is G2, G1.5, and G0, as shown in Figure 12b. Extending the grinding time not only reduces the porosity of the ITZ but also improves the hydration process of the cementitious material in the ITZ. In contrast to the pore structure test results obtained in the MIP test, the extended grinding time of IOTs increased the pore volume of the concrete matrix and deteriorated the pore structure. Some scholars refer to the ITZ as the bridge between the aggregate and the matrix [57]. Even if the matrix or aggregate has excellent properties, the overall performance of concrete will be reduced due to the poor ITZ, which cannot transfer high stresses from the matrix to the aggregate. This reveals the reason for the inconsistency between matrix pore structure properties and compressive strength.

Figure 13 shows the trend of porosity and unhydrated rate in the ITZ of concrete with dopant admixture. The porosity of the ITZ decreases with increasing SCMs content, as shown in Figure 13a, which is the opposite of the trend of the porosity of the concrete matrix. This is because the total amount of water and cementitious materials is certain. The addition of SCMs can improve the ITZ by exerting a volcanic ash effect and a filling effect, in which a certain percentage of fine particles can be closer to the aggregate and reduce the sidewall effect of the aggregate. At the same time, the auxiliary gelling material can act as a nucleation point to reduce the orientation of calcium hydroxide crystals in the ITZ [18,19]. The total amount of colloidal material in the matrix decreases, the water–cement ratio increases, and therefore the matrix porosity increases. As shown in Figure 13b, the unhydrated rate of C20 is lower than that of C0, whereas the unhydrated rate of C30 is greater than that of C0. This indicates that the admixture enhances the mechanical properties of the ITZ by promoting the hydration process the ITZ. However, when the dosage exceeds 20%, the promotion turns into inhibition. Although C30 has lower porosity in the ITZ, due to its higher unhydration rate, the cohesion between particles in the ITZ is poor, so the strength is lower than that of C0 and C20. C20 has better ITZ performance than C0, but the strength is lower than that of C0, so it is speculated that part of the matrix is damaged due to the deterioration of the matrix pore structure by the admixture.

To further support the results of the BSE image analysis, EDS line sweeps were performed on the gelatinous material on the outside of the aggregate from the ITZ, and more detailed information on the variation of the gelatinous material along the line element concentration on the outside of the ITZ was obtained by the EDS line scan. The Ca/Si ratio of each specimen is listed in Table 4, and when 0.8 ≤ Ca/Si ≤ 2.5 is judged to be a hydrate rich in C-S-H [58]. For G0, the Ca/Si of G1.5 and G2 decreased, indicating that the improvement of the ITZ was more significant after the grinding of IOTs. Compared with C0, C20 and C30 have different degrees of decrease, i.e., the degree of Ca/Si decrease is more obvious with the increase of dosing. It indicates that the auxiliary cementing material can consume CH to generate C-S-H to improve the pore structure of the ITZ and reduce the Ca/Si at the ITZ. The EDS analysis results are consistent with BSE [59].

## 4. Conclusions

In this study, the composite SCMs system was prepared by IOTs-PS-SS, and the effects of the type of SCMs, IOTs admixture, IOTs grinding time, and SCMs admixture on the concrete properties were investigated. The effects of mechanical grinding on the activity of IOTs were explored by XRD and XPS. The compressive strength test, MIP, BSE, and EDS were used to test the compressive properties, pore structure, ITZ, and elemental distribution of hydration products of concrete, which provide a reference for the application of building materials in the full particle size range of IOTs. The main conclusions are as follows:

1. Compared with using IOTs alone as SCMs, there are significant advantages of using SCMs in the compounding system. PS makes up for the low activity of IOTs, and the synergistic mechanism between SS and PS can further enhance the compressive properties of concrete. Changing the fineness of IOTs can improve the compressive properties of concrete thanks to its increased activity.
2. The pores of the ITZ of concrete were optimized after the introduction of composite SCMs. When the dosage is increased by 20% or the grinding time of IOTs is increased, it can reduce the porosity of the ITZ and promote the hydration of the particles within the ITZ, thus reducing the calcium–silica ratio and achieving the purpose of enhancing the ITZ.
3. Composite SCMs can deteriorate the matrix pores of the concrete. The increase in pore volume and the coarsening of pore structure are mainly manifested by the increase in the amount of SCMs or the change in the fineness of IOTs. This degradation will be aggravated by the possibility of inducing matrix damage, which will lead to the loss of concrete strength. However, the strength loss due to matrix pore deterioration is within the acceptable range since concrete mostly suffers from interface damage. Therefore, the recommended value of compound SCMs admixture is 20–30%.
4. The introduction of composite SCMs makes concrete suffer from a common defect, i.e., lower early strength than normal concrete. The mechanism behind this phenomenon and improvement measures will be the focus of subsequent research.

## Figures and Tables

**Table 1:** Chemical compositions of IOTs, PS, steel slag, and cement (w/%).

Composition	SiO_2_	Fe_2_O_3_	CaO	MgO	Al_2_O_3_	K_2_O	Na_2_O	P_2_O_5_
IOT	62.26	14.37	7.78	6.33	4.78	1.40	1.34	—
PS	39.08	1.14	47.45	2.90	3.94	0.87	0.60	2.34
SS	15.2	27.54	42.65	6.05	2.53	0.06	0.02	1.97
Cement	22.50	3.43	66.30	0.83	4.86	0.43	0.22	—

**Table 2:** Mix proportions of concretes (kg/m^3^).

Samples	Cement	Water	SCMs			IOTs-FA	IOTs-CA	WR	IOT Grinding Time
			IOT	PS	SS				
M1	294	185	126	0	0	740	1110	4.5	2 h
M2	294	185	63	63	0	740	1110	4.5	2 h
M3	294	185	63	32	32	740	1110	4.5	2 h
I15	294	185	63	32	32	740	1110	4.5	2 h
I10.5	294	185	44	41	41	740	1110	4.5	2 h
I6	294	185	25	50	50	740	1110	4.5	2 h
I1.5	294	185	6	60	60	740	1110	4.5	2 h
G0	294	185	25	50	50	740	1110	4.5	0 h
G1.5	294	185	25	50	50	740	1110	4.5	1.5 h
G2	294	185	25	50	50	740	1110	4.5	2 h
G2.5	294	185	25	50	50	740	1110	4.5	2.5 h
C0	420	185	0	0	0	740	1110	4.5	0
C10	378	185	8.5	17	17	740	1110	4.5	2 h
C20	336	185	17	34	34	740	1110	4.5	2 h
C30	294	185	25	50	50	740	1110	4.5	2 h

**Table 3:** Specific surface area of IOTs at different grinding times.

Grinding Time/h	0	1.5	2	2.5
Specific surface area/m^2^·kg^−1^	646	1290	1587	1311

**Table 4:** Ca/Si molar ratio estimated by EDS line analysis.

	G0	G1.5	G2/C30	C0	C20
Ca/Si	1.76	1.74	1.14	1.81	1.42
